# A Bill for an Act to Regulate the Practice of Dentistry in Wyoming

**Published:** 1893-04

**Authors:** 


### LEGISLATION.

### A Bill for an Act to Regulate the Practice of Dentistry in Wyoming.

*Be it enacted by the Legislature of the State of Wyoming:*

Section 1. It shall be unlawful for any persons to practice dentistry or dental surgery in the State of Wyoming without first having received a diploma from a reputable Dental College or University, duly incorporated and established under the laws of some one of the United States or some foreign government, which is recognized as such by the National Association of Dental Examiners: *Provided*, that nothing in Section one of this Act shall apply to any bona fide practitioner of dentistry or dental surgery in this State at the time of the passage of this Act; and *Provided*, That nothing in this Act shall be so constructed as to prevent physicians or surgeons from extracting teeth.

Sec. 2. Every person who shall hereafter engage in the practice of dentistry or dental surgery in this State shall file a copy of his or her diploma with the County Clerk of the County in which he or she resides, which copy shall be sworn to by the party filing the same, and the Clerk shall give a certificate with the seal of the County attached thereto, to such party filing the copy of his or her diploma, and shall file or register the name of the person, the date of the filing, and the nature of the instrument, in a book to be kept by him for that purpose.

Sec. 3. Every bona fide practitioner of dentistry or dental surgery residing in this State at the time of the passage of this Act, and desiring to continue the same, shall within sixty days after the passage of this Act, file an affidavit of said facts as to the length of time he or she has practiced in this State, with the County Clerk of the County in which he or she resides, and the said Clerk shall register the name of and give a certificate to the party filing the affidavit, in like manner and of like effect as hereinbefore provided.

Sec. 4. All certificates issued under the provisions of this Act shall be prima facie evidence of the right of the holder to practice under this Act.

Sec. 5. Every person violating the provisions of this Act shall, upon conviction thereof, be deemed guilty of a misdemeanor, and punished by a fine of not less than fifty dollars, nor more than two hundred dollars, for each and every offense, or be imprisoned in the County jail for sixty days, or both fine and imprisonment, at the discretion of the court, and all fines collected shall belong to and be paid into the common school funds of the County where the offense was committed.

Sec. 6. Any person who shall have filed his or her affidavit or diploma, as required in Sections two and three of this Act, in one County and remove to another County, shall, before entering upon the practice of his or her profession in such last named County, procure a certified copy of the record of his or her former registry, and cause such transcript to be filed and recorded in the dental register of such County in which he or she has removed.

Sec. 7. This Act shall take effect and be in force from and after its passage.

Was approved by the Governor and became a law on February 18th, 1893.

